# Supplementary material for: Are carbon emissions trading and green financial instruments synergistic? -Comprehensive quantitative research based on content analysis
Source: PLoS One. 2024 Mar 7;19(3):e0298601. doi: 10.1371/journal.pone.0298601 (PMC10919601; doi:10.1371/journal.pone.0298601)
Supplement: S1 Data — (ZIP) [file pone.0298601.s001.zip › Data/地级市Z_F工作报告.zip!/░▓╗╒/╗┤─╧╩╨/└┤╫╘╣1⁄2╓┌║┼í╛┬φ┐╦╩2╛▌═°í┐_2015.docx]

各位代表：
    现在，我代表市人民政府，向大会报告工作，请予审议，并请市政协委员和其他列席人员提出意见。
    一、2014年工作回顾
    刚刚过去的2014年，是淮南发展进程中极为困难的一年。面对经济下行、结构性矛盾和重大事件影响等复杂形势，全市人民在省委省政府和市委的坚强领导下，认真贯彻党的十八大、十八届三中、四中全会和习近平总书记系列讲话精神，坚持事不避难、勇于担当，积极化解风险，奋力克难攻坚，保持了经济运行安全和社会大局稳定。初步核算，全市地区生产总值780亿元，规上工业增加值400亿元，财政收入125.8亿元，固定资产投资760亿元，进出口总额4.4亿美元，社会消费品零售总额314.5亿元，城镇、农村常住居民人均可支配收入分别为26170元、10400元，在深化改革、推动转型、改善民生的进程中迈出了坚实的步伐。
    一年来，重点做了以下八个方面工作：
  （一）强化综合施策，发展基础不断夯实。全面落实国务院和省政府一系列政策措施，着力优化存量、扩大增量，确保经济运行安全、风险可控。夯实实体经济基础。着眼于为企业松绑减负，减免增值税、营业税、企业所得税等税负14.2亿元。出台金融支持企业直接融资奖励办法等文件，直接融资增长167.3%，8家企业分别在“新三板”和区域性股权交易市场挂牌。扎实推进注册资本登记制度等改革，新增各类市场主体24720家，净增“四上”企业265家。梳理解决284家民营企业涉及的土地、规划、产权等制约发展的历史遗留问题，民营经济增加值占地区生产总值的比重提高1个百分点。夯实项目投资基础。坚持经济工作项目化、项目工作责任化，建立重点项目包保和调度机制。新开工亿元以上项目65个、续建184个、竣工78个，累计完成投资409亿元。中安煤化一体化、中国移动（安徽）数据中心等项目加快推进，朱集西矿等项目基本建成，全市电力装机容量达1204万千瓦。夯实居民消费基础。积极培育网络、信息、文化、旅游等新的消费增长点。房地产新开工400万平方米、竣工150万平方米。旅游业实现总收入70亿元。江淮云产业基地获批省电子商务示范园区，田家庵跻身省级服务业综合改革试点区。夯实产业转型基础。强化工业经济运行调度，致力优化产业结构，加快培育接续替代产业，非煤产业增加值占规上工业增加值的比重提高4.8个百分点。着力拓展发展空间和环境容量，淘汰一批落后产能，依法关闭退出小煤矿15对、非煤矿山企业4家，彻底告别了小煤矿百年开采史，全面迎来了现代化大矿井时代。
  （二）强化改革创新，发展活力不断增强。从制约经济发展的关键环节入手，转职能、补短板，抓开放、促创新。重点领域改革加快推进。完成市政府权力清单及权力运行流程图编制，并予以公布。对176项行政审批事项进行清理，保留市级行政许可55项。县区工商、质监、食品药品监管体制改革完成。基层医药卫生体制综合改革稳步推进。资金、资产和土地“三项清理”工作扎实开展。凤台农村土地确权颁证试点工作有序推进，“粮食银行”模式得以推广。科技创新能力持续增强。省级创新型城市试点全面启动。全社会研发经费投入15亿元。授权发明专利236件。制定修订行业、地方标准10项。“两化融合”指数提高4个百分点。中科院淮南新能源研究中心等平台加快建设，全国防爆电气知名品牌示范区获批筹建，煤化工产品质检中心通过国家评审。对外开放水平逐步提升。积极融入长三角、中原经济区，加快合肥经济圈一体化进程，与塞尔维亚武贝市建立友好城市。园区承载功能不断提高，安徽（淮南）新型煤化工基地、国家级淮南经开区、淮南高新区加快建设。强化精准招商和产业链招商，全年利用外商直接投资2亿美元，引进内资600亿元。支持本土企业立足淮南，开展对外合作。合肥海关驻淮南办事处获批成立，为本地企业外向发展提供通关便利。
  （三）强化城市功能，城镇化步伐不断加快。坚持以人为本，促进公共服务均等化,新增建成区面积2.6平方公里，城镇化率达66.9%。城乡规划加快编制。统筹经济社会发展规划、土地利用总体规划、城乡总体规划，开展“多规合一”试点。各类规划加快完善，城乡规划体系更加科学合理，规划的前瞻性、约束性和严肃性进一步增强。山南新区建设稳步推进。按照“三年大见成效，五年基本建成”的发展要求，重新启动一批战略合作和重点项目建设，核心区路网、公交等设施加快完善，3所学校、2所幼儿园建成投用，安理大新校区、职教园区等群众关注的项目全力推进。安居工程建设保障力度加大。全面完成保障性安居工程年度建设任务，开工59185套、基本建成13395套。利用住房公积金贷款3.1亿元，支持保障性安居工程建设。扎实推进采煤塌陷区异地搬迁工程，竣工安置项目4个。加强安置区建设协调调度，着力解决征迁户安居问题。城市基础设施加快完善。西部城区5条道路改造完成，3座淮河大桥加快建设，新改建各类地下管网180公里，龙王沟城市排涝工程全面竣工。交通网络加快构建，济祁高速公路淮南段、九龙大道等项目开工建设。“三线三边”环境综合整治扎实开展，违法建设、超载超限专项整治取得积极成效。全国智慧城市试点工作全面启动。
    （四）强化“三农”基础，农村面貌不断改善。积极整合各类支农政策和资金，“三农”支出34.2亿元，增长5.2%。现代农业稳步发展。粮食总产139.4万吨，实现“十一连丰”。农业规模化经营力度加大，新增国家农业产业化示范园1家，新增流转、托管土地19.3万亩。新型经营主体加快培育，新增专业合作社492家、家庭农场211家。生态农业、特色农业加快发展。农村面貌有效改善。建成美好乡村中心村48个，3个���跻身全国重点镇。完成农村危房改造和农房建设5496户，实施2010户渔民上岸安居工程。完成农村危桥改造18座、农村清洁工程11个，改造省道、县乡道路86.4公里。解决10.7万人安全饮水问题。全年脱贫9200人。农业基础设施建设扎实推进。重点平原洼地和中小河流治理工程、大型泵站更新改造和小型水利改造提升工程加快实施。建立耕地保护动态巡查机制，土地违法整改深入推进。整治土地24.7万亩，连续14年实现耕地占补平衡。推进淮河沿线综合整治，严厉打击非法采砂行为。
  （五）强化公共服务，民生福祉不断增强。优先保障民生资金支出，全面完成33项民生工程。全年民生支出118亿元，占财政总支出的80.8%。就业形势总体向好。城镇新增就业4.7万人，转移农村劳动力2.3万人，大学生、退役军人和失地农民等群体实现稳定就业。社会保障体系不断完善。城镇职工基本养老金、城乡低保、五保供养标准均提高10%以上，医疗救助报销标准增长9%。建立城镇医保大病保险制度，新农合参合率达101.2%。文化强市扎实推进。市文化艺术中心等项目加快建设，文化惠民工程深入实施。5家企业进入全省民营文化企业百强，1家企业成为国家文化出口重点企业。《淮南子传奇》第二季喜获第27届中国电视金鹰奖。各项社会事业加快发展。出台进一步加强中小学教师队伍建设的实施意见。23所义务教育标准化学校和市体育学校基本建成，标准化学校覆盖率达90.3%。全民健身服务体系日趋完善，圆满完成第十三届全省运动会等参赛任务。卫生和计生职能加快整合，人口自然增长率7.22‰。第三次全国经济普查工作全面完成。地震、气象、档案、民族、宗教和地方志工作不断加强，妇女、儿童、老龄和残疾人事业取得新的成效。
  （六）强化节能减排，生态环境不断改善。推进绿色低碳循环发展，逐步改善环境质量。重点治理工程扎实推进。投入污染治理资金10.5亿元。实施节能和综合利用项目50个，西部生活垃圾焚烧电厂并网发电，淮化工业废水末端治理等重点工程基本建成，提前完成“十二五”火电行业脱硫脱硝减排工程建设任务。单位生产总值能耗下降1.5%左右，主要污染物减排年度任务全面完成，淮河流域水污染防治年度考核全省第一。全面完成大气污染防治任务。加强秸秆禁烧与综合利用，拆除小型燃煤锅炉377台，治理大型工业燃煤锅炉15台，依法关停超标排污企业及作坊50家。PM10平均浓度较上年下降7%，在全省位次提升2位。大气环境二级以上天数比上年增加14天。有效开展生态修复。坚持不懈对采煤塌陷区进行生态修复和综合治理。启动关闭退出小煤矿、采石场覆土复绿工作。完成成片造林近10万亩，新建森林长廊80.1公里，省级森林城镇和森林村庄、省级生态县和生态乡镇创建取得积极成效。
  （七）强化治理创新，平安建设不断深入。推动社会治理重心下移，信息化管理和社会化服务得到加强。平安淮南建设稳步推进。社会治安防控体系逐步建立，视频监控系统加快完善。全面开展大巡防工作，成功破获一批大要案，社会治安持续向好。强化应急平台和队伍建设，有效处置各类突发事件。加强矛盾纠纷排查调处，基层综治维稳信访工作中心（站）全面建成，群防群治工作得到加强。稳妥处置重大安全事件。教训深刻的“8·19”民营小煤矿爆炸事故发生后，市委市政府和淮南矿业集团坚持现场指挥、科学救援、确保稳定，凝心聚力奋战52天，依法完成善后安抚、闭矿处置等工作。严防淮矿物流经营风险蔓延，保持区域性金融稳定。社会治理基础进一步夯实。村民自治能力不断加强，村委会换届选举工作顺利完成。落实领导干部接访、下访、约访、回访和定点帮扶机制，一批群众关心的问题得到解决。建成网上信访信息系统，逐步实现信访业务网上流转，最大限度地把矛盾和问题预防在发生之前、化解在萌芽状态、处置在初始阶段。举行首个烈士纪念日公祭烈士活动，国防动员、人民防空和双拥工作进一步加强。
  （八）践行“三严三实”，政府自身建设不断加强。坚持从自身做起，着力强化政府公共服务、市场监管和社会治理职能。群众路线教育实践活动扎实开展。聚焦“四风”等突出问题，切实加大整改力度，着力解决联系服务群众“最后一公里”问题，逐步完善作风建设长效机制。依法行政不断加强。通过宪法日的宣传和教育，弘扬宪法精神，建设法治政府。按时办结130件市人大代表议案建议和372件市政协委员提案，提请审议地方性法规3件，制定政府规章2件，在全国率先出台推进依法行政办法。实行规范性文件合法性审查等制度，重大事项决策行为更加规范。不断深化政务公开、网络问政，政务服务更加便捷高效。廉政建设扎实推进。严格执行中央八项规定和国务院“约法三章”，不断加大反腐倡廉工作力度，全市“三公”经费压缩23.8%。加强审计监督，推进审计全覆盖，审计调查单位637家，促进增收节支4.6亿元。强化行政监察，给予政纪处分112人，塑造了为民务实清廉的政府形象。
各位代表！过去的一年，面对经济下行的巨大压力，我们多措并举、防微杜渐，化解了各种潜在风险；面对各种事件的接踵而至，我们沉着应对、主动作为，确保了社会大局稳定。这是全市广大干部群众同心协力、奋力拼搏的结果。在此，我代表市人民政府，向全市人民，向驻淮部队、武警官兵和政法干警，向各民主党派、各人民团体和各界人士，向关心支持淮南发展的同志们、朋友们，向在淮创业的投资者、建设者，表示衷心的感谢和崇高的敬意!
    同时，我们也清醒地看到，发展中还存在不少困难和挑战。一是煤炭价格低位徘徊，非煤产业发展不足，县区、园区、民营经济基础薄弱，转型发展任重道远。二是城市基础设施建设欠账较多，公共服务设施配套不足，征迁、安置和城市管理任务较重。三是土地、资金、人才等要素制约突出，项目落地困难，投资环境不优。四是少数干部进取意识不强，法治意识淡薄，为官不为、为官乱为的现象仍有发生。对此，我们将高度重视，切实加以解决，决不辜负全市人民的希望和重托。
    二、2015年工作思路和重点
      今年是全面深化改革的关键之年，是全面推进依法治市的开局之年，也是实施“十二五”规划的收官之年,做好今年工作意义重大。当前，世界经济仍处在深度调整期，国内经济进入速度变化、结构优化、动力转换的新常态。新常态面临新挑战，也蕴含新机遇。我们必须坚定战略定力，提振发展信心，适应新常态，抢抓新机遇，开创新局面。
     今年工作的总体要求是：全面贯彻党的十八大、十八届三中、四中全会和中央、全省经济工作会议精神，坚持稳中求进工作总基调，坚持以提高经济发展质量和效益为中心，主动适应经济发展新常态，转方式、调结构、促改革、重创新、抓招商、防风险、惠民生，促进经济平稳健康发展、社会和谐稳定。
     在立足实际、综合研判的基础上，提出主要预期目标：地区生产总值增长5%，财政收入增长3%，固定资产投资增长10%，城镇和农村常住居民人均可支配收入分别增长6%、8%，城镇新增就业4万人以上，完成省下达的节能减排目标任务。
    为实现以上目标，关键要把握以下四点。
    一要牢牢把握全面建成小康社会这个总体目标。全面建成小康社会，既要体现经济发展速度、质量和效益相统一，更要促进社会和谐、文化繁荣、生态宜居、政治稳定相协调，推动质量效率型集约增长。紧扣民生幸福主旨，守住环保和就业两个底线，彰显公平正义，实现群众利益最大化、公共服务最优化。
    二要牢牢把握全面深化改革这个核心动力。改革只有进行时，没有完成时。我们必须把改革贯穿于经济社会发展始终，进一步激发市场活力和社会创造力。必须遵循经济规律、自然规律和社会规律，坚持问题导向、目标指向，促进简政放权、放管结合，在全市营造大众创业、万众创新的浓厚氛围。
    三要牢牢把握全面依法治市这个基本准则。坚持在法治轨道上开展工作，运用法治思维和方式化解社会矛盾，更好统筹社会力量、平衡社会利益、调节社会关系、规范社会行为。着力推进政府机构、职能、权限、程序、责任法定化，建立权责统一、运行高效的依法行政机制。 
    四要牢牢把握全面转型这个鲜明主题。唯有转型，方能行稳致远。要做精做优煤电化产业链、做大做强非煤产业群，为支柱产业挖潜增效、为主导产业嫁接延伸、为高成长性产业培土施肥。要加快产城融合化、城乡一体化、合淮同城化，实现转型发展、开放发展、安全发展、和谐发展、廉洁发展。
    实现以上目标，要重点做好以下八个方面工作。
  （一）把深化改革作为活力源泉，着力增强发展动力
解决经济社会发展中的矛盾和问题，要求全面深化改革，准确把握改革发展稳定的平衡点，释放更多改革红利。
    通过改革释放体制机制活力。推进政府机构改革，建立精干高效的政府组织体系。深入推进简政放权，积极做好行政审批事项的承接、精简、下放、监管等工作。开展行政审批代办服务，推行网上审批。全面推行权力清单、责任清单和涉企收费清单制度，完善政府权力公开运行、动态监管机制。建立跨年度预算平衡机制和预决算公开机制。改制市级投融资平台。加快事业单位分类改革，开展事业单位法人治理结构试点工作。深化教育综合改革，组建职业教育集团，推进中小学教师无校籍管理。完善行政执法体制，建立综合执法机制。统筹推进基层医药卫生体制、公务用车制度、社区管理体制等改革，进一步释放体制机制活力。
    通过改革释放市场要素活力。深化商事制度改革，全面推进“三证合一”、“先照后证”、登记注册全程电子化管理改革。坚持放开竞争性领域，积极推广运用政府与社会资本合作（PPP）模式，推进投资主体多元化。强化政府性债务管理，完善政府投资项目招投标体制和决算审计改革。建立健全中小企业贷款“助保金”制度，建立完善市县（区）政策性担保体系。巩固并推广农村金融综合改革成果，大力发展普惠金融。加大非法集资打击力度，建立金融机构风险处置协调机制。坚持土地统一收储、统一出让、统一考核，确保土地等要素跟着项目走。加快户籍制度改革，有序推动农村转移人口市民化。
    通过改革释放市场主体活力。支持在淮省属企业改革，促进股权多元化、运营专业化。推进市属企业改革，加快企业上市、兼并、重组步伐。继续解决制约企业发展的历史遗留问题，让市场主体轻装上阵。推进国有粮食购销企业、供销社、集体林权制度配套改革，完成农村土地确权登记颁证任务。做好经营性资产清查，巩固资金、资产和土地清理成果，规范国有资产租赁管理行为。加强社会组织改革创新，完善社会组织培育扶持政策。
  （二）把稳定增长作为重中之重，着力提升发展质量
    坚持定向施策、精准发力，积极挖掘和培育新的增长点，实现有速度、有质量、有效益的发展。
扩大有效投资。充分发挥投资的牵动作用，完善项目储备库，健全项目投资联合审查、协调调度、政策支撑、要素保障机制。在主导产业、基础设施、节能环保、社会事业等重点领域，集中力量谋划一批、推进一批、竣工一批重大项目，力争全年完成投资830亿元。进一步优化投资结构，持续推进项目建设提质提效。发挥中原经济区、淮河生态经济带等战略平台优势，申请纳入全国资源型城市可持续发展试点市，争取更多项目和政策支持。
    促进城乡消费。坚持把发展服务业与促进消费结合起来，完善产业体系，推进业态创新，力争服务业增加值增长6%以上。适应消费新形势，促进城市商圈升级发展，满足群众个性化、多样化消费需求。培育大众消费新的增长点，拓展信息、家政、文化、旅游、健身等消费。加快现代物流业发展，谋划快递产业园、专业物流中心等建设，促进电商、网购、快递融合发展。大力发展检验检测认证、金融保险、商务咨询、技术服务等产业。健康发展房地产业，全年开工200万平方米，竣工120万平方米。
    做强县域经济。健全支持县区发展的相关政策，理顺市县区财权事权关系，赋予县区更多自主权。引导县区立足区位条件、资源禀赋、产业基础，不断提升产业集中度和首位度。支持淮河以北地区做精做优煤电化产业链，提升煤电化产业联营和配套水平，因地制宜发展现代农业和乡村旅游业，综合治理采煤塌陷区，提升煤电化一体、城矿乡一体发展水平。支持淮河以南地区做大做强非煤产业群，加快发展战略性新兴产业、现代服务业，提升产城一体、旅城一体和新型城镇化水平。完善县区发展科学评价体系，提升工业经济、园区发展、城乡建设等考核权重，激发县区发展动力和活力，形成淮河两岸整体联动、互补发展新格局。
    夯实园区载体。逐步完善园区体制机制，扩大园区发展自主权，增强园区造血功能。制定支持园区发展的实施意见，明确园区发展定位和规划布局，建立科学考核机制。坚持产业集中、企业集群、土地集约、要素集聚，加快园区基础设施建设，引导发展要素向园区倾斜。大力提升园区服务配套能力，营造引进企业、留住人才的创新创业环境。坚持市经开区产城一体发展，打造百亿产业园区。加快现代煤化工产业园公用工程和基础设施建设，力争烯烃装置基本建成，确保动力中心、净水厂、变电所和码头投入运行。完善市高新区总体规划和专项规划，力争引进电子商务和信息服务类企业30家。统筹园区招商、发展和为企服务，坚持不懈做大园区经济，持之以恒提升园区在全市经济中的份额。
   （三）把调整结构作为主攻方向，着力促进发展转型
    坚持立足煤、延伸煤、不唯煤、超越煤“四煤”发展思路，促进产业发展提质增效升级，力争非煤产业比重稳步提升，现代服务业实现量增质优。
    大力扶持产业发展。编制产业发展规划，建立扶持发展政策体系，设立产业发展引导基金，打造面向未来的产业支撑。巩固提升煤炭、电力两大支柱产业，加快两淮能源基地规划编制，推进杨村矿建设，开工建设1000千伏特高压北环线，实现平电三期并网发电，做好潘集电厂、低热值煤电厂等项目前期工作。加快发展新型煤化工、汽车及装备制造、新一代信息技术三大主导产业，加快中安煤化一体化、凤台煤制天然气、江淮云产业基地、电子商务产业园、陕汽配套产业园等重点项目建设。大力培育文化旅游、现代医药、光电新能源、绿色有机农产品加工四大高成长性产业，着力推进旅游景区、生物医药科技园、光电产业园、国家级农业产业化示范区等建设。
    实施创新发展战略。拓宽研发经费投入渠道，鼓励企业建立联合发展机制，促进创新多主体协同、多要素联动、多领域合作。围绕新技术、新机制，加快市高新区科技研发中心、科技研发孵化平台、淮南新能源研究中心等建设。提高发明专利申请量、拥有量及转化率。新建院士工作站3个。改造提升传统产业，完成技改投资200亿元。加快全国智慧城市试点、省级创新型试点市建设，力争第九次荣获全国科技进步先进市。
    提升对外开放水平。大力推进招商引资、招才引智工作，建立健全项目落地约束和保障机制，坚持“一个投资项目、一个牵头领导、一个工作小组”专班推进，营造全域招商、全员招商、全面招商的良好氛围。紧盯长三角、珠三角、环渤海等重点区域，强化精准招商。制定重点产业招商规划，注重产业链招商。积极实施“走出去”战略，加快外经贸转型升级。着力培育外贸经营主体，进出口总额实现稳定增长。加快申报淮南商检正式机构、公共型保税仓库，不断提升外向型经济比重。
积极培育市场主体。落实扶持小型微型企业健康发展等措施，着力培育更多有活力、有实力、有竞争力的中小微企业。进一步放宽登记条件，加大市场主体培育力度，完善支持“四上”企业发展的措施。进一步加大金融支持力度，推进创业园和创业孵化基地建设。净增民营企业3000家，新增个体工商户1万家。培育10家高成长性的战略性新兴企业，建设智慧企业120家，���增“双软”认定企业5家。发挥企业创新转型升级的引领作用，营造支持创业、鼓励创新、宽容失败的良好氛围。
  （四）把“三农”发展作为坚实基础，着力夯实发展根基
    加快转变农业发展方式，推进城乡一体化进程，让农业成为有奔头的产业，让农民成为专业化的职业，让农村成为美丽的家园。
    提高农业综合生产能力。落实国家粮食安全战略，力争粮食总产稳定在120万吨以上。加强粮食仓储设施建设，扩大粮食收储能力。深入实施“菜篮子”工程，提高农产品保障水平。加强农产品标准化建设，创建农业标准化示范区10个、省级优质农产品生产基地2个。建成农产品质量安全检验检测中心。实施最严格的水资源管理制度。抓好农田水利基本建设，加快大型泵站更新改造，开工建设西淝河等沿淮洼地应急工程，积极推进引江济淮工程，建成世行贷款洼地治理项目，提高防洪排涝和抗旱保收水平。
    创新农业生产经营体系。出台支持现代农业发展的政策措施，推动三次产业深度融合。坚持用工业的理念抓农业，大力提高农业现代化装备水平，力促规模以上农产品加工业产值稳定增长。推动农业适度规模经营，加快43个土地托管服务点建设。加快培育新型经营主体，新增农民专业合作社100家、家庭农场50家。加强农民工职业技能培训，培育新型职业农民。
    推进美好乡村建设。实施农村集镇建设规划改造，启动美丽小镇建设试点，建成一批美好乡村中心村。加强农村基础设施建设，新建和改造国省干线48公里、县乡道路40公里，健全城乡一体化养护机制。深入推进“三线三边”环境综合整治，改善农村居住环境。扎实开展精准扶贫，实施扶贫开发和江淮分水岭综合治理项目，力争全年脱贫7000人。
  （五）把宜居宜业作为功能特质，着力推进城市建设
    坚持发展淮南、呼应省会、强化自身、承接辐射，促进山水林城融为一体、宜居宜业相得益彰，打造合肥经济圈副中心城市。
    彰显城市特色。发挥“三山鼎立、三水环绕”的自然优势，编制城市风貌特色等专项规划，启动八公山、焦岗湖旅游开发规划编制，完成淮河岸线资源开发利用规划、淮河港总体规划。加快合淮一体化总体规划编制，完成凤台县和毛集实验区总体规划编制，完善开发区控规及各类专项规划。加快“多规合一”试点，编制“十三五”规划，推进“规划一张图”建设。
    完善城市功能。推动山南由新区向新城转变，以政务区、高新区、高铁南站、职教园区为重点，完成职教中心和工业学校整合搬迁，推进实验中学新校区、市妇幼保健院新院、引大别山优质水源工程等项目建设。新改建各类管网125公里。推进亚行贷款城市水系综合治理工程。按照“内畅外联、便捷安全”的要求，启动商杭高铁淮南段建设、合淮路淮南段改造，加快济祁高速公路淮南段、九龙大道等建设，推进跨淮河大桥建设，完成城市部分道路、铁路下穿等工程。
    建设宜居宜业城市。推进老旧小区改造，加快安置区建设，实施西部环境整治提升工程。开展城镇园林绿化提升行动，实施城市绿道示范段工程，建成30公里城市绿道，新增和改造绿地430万平方米。制定公共交通扶持政策，推动公共交通事业发展。推进环卫体制改革和志愿服务制度化，加强占道经营、乱停乱放、道路抛洒等顽症治理。创新城市管理考核体系，不断提升城市管理水平。 
  （六）把改善民生作为根本主旨，着力促进社会和谐
    坚持民生为上，更加注重保障基本民生，更加关注困难群体，确保人民群众安居乐业。
    加大为民办实事力度。继续实施民生工程，完善民生工程考核评价办法和建后管养机制。突出为民办实事。开工建设棚户区改造房屋10234套、公租房1449套。开工建设采煤塌陷区安置房4200套，搬迁1.2万人。完成2500户农房建设、1500户农村危房改造任务。解决10.8万人安全饮水问题。扎实做好关闭退出小煤矿职工就业培训等工作。
    加快社会事业发展。继续实施第二期学前教育三年行动计划，力争入园率达87.6%。争创国家义务教育均衡发展县区，义务教育标准化率达97.3%。实施卫生惠民工程，推进基本公共卫生服务项目建设，强化卫生应急和重大疾病防控。广泛开展全民健身运动，促进群众体育和竞技体育全面发展。落实人口目标管理责任制，综合治理出生人口性别比偏高问题，人口自然增长率控制在8.5‰以内。加快社会服务管理信息化建设。推进农村为民服务全程代理工作。支持少数民族聚居地区优先发展，巩固提升民族宗教工作。做好外事侨务、妇女儿童、残疾人、档案、地方志和机关事务管理工作，支持地震、气象、防灾减灾事业发展，发挥工会、共青团、妇联等人民团体桥梁纽带作用。
    完善社会保障体系。加大创业就业政策支持力度，创新大学生就业就学人才培养模式，统筹做好失地农民等群体就业。培训各类人员4.9万人。强化社会保障和养老服务，完善城乡居民基本医疗保险和大病保险制度，提高医疗救助水平。抓好各类社会保险扩面提标，推动老农保和城乡居保有效衔接。推进社会救助制度改革，分别提高城乡低保、五保供养标准10%以上。居民消费价格控制在3%左右。
  （七）把历史文化作为城市灵魂，着力提升城市内涵
    坚持经济效益和社会效益相统一，持续推动文化繁荣发展，增强文化创造力和竞争力。
践行社会主义核心价值观。深入开展社会主义核心价值观、中国梦的宣传教育，推进公民道德和精神文明建设，凝聚全市人民团结奋进的强大正能量。宣传淮南好人好事，讲好淮南故事。广泛开展“讲文明树新风”活动，扎实做好文明单位系列创建。加强社会信用体系建设，培育诚信文化，打造诚信社会，让失信者难以立足、让守信者一路绿灯。
    繁荣发展文化事业。推进国家历史文化名城创建。加强文物和非物质文化遗产保护。实施文化惠民工程，加快市文化艺术中心、历史文化街区建设，推动“大地欢歌”等群众性文化活动开展。加大文化人才培养力度，营造文艺创作的良好环境。发展哲学社会科学、文化艺术、新闻出版、广播电视等事业，提高城乡居民的文化生活质量。
    推进文化旅游融合发展。坚持保护文化、挖掘文化、创造文化相结合，推进一批文化创意产业园建设。实施文化精品战略，打造具有地方特色、有竞争力的文化产业，实现文化产业增加值增长20%。建立文化旅游发展协调机制，推动文化旅游与新型工业化、信息化、城镇化和农业现代化相结合，积极发展乡村休闲游、历史文化游、山水特色游、生活体验游。加快景区扩容升级，打造八公山和焦岗湖两大核心旅游区，启动景区5A级创建。全年旅游业总收入增长10%以上。
  （八）把创新治理作为重要抓手，着力优化发展环境
    坚持保护环境就是保护生产力、改善环境就是发展生产力，促进社会环境、投资环境、生态环境同步优化。
    建设平安淮南。健全依法维权和化解纠纷机制，引导和支持群众理性表达诉求、依法维护权益，畅通群众利益协调、权益保障渠道。落实信访维稳工作责任制，创新网上信访、逐级走访、诉访分离机制。完善社会风险评估机制，提高规范化、科学化水平。加强行业性、专业性人民调解组织建设，依法解决好群众最关心最直接最现实的利益问题。持续推进立体化、数字化社会治安防控体系建设，依法严厉打击各类违法犯罪活动。完善应急处置快速协调联动机制，增强应对各类突发事件的能力。加强国防动员、后备力量、国防教育和人民防空工作，推动军民深度融合发展，争创全国双拥模范城“七连冠”。
    优化投资环境。建立问政质询机制，对纪律松弛、组织涣散、作风转变缓慢的单位，加强问政监督。建立效能问责机制，坚决整治政令不畅、效率低下、贻误工作、不负责任等问题。建立综合整治机制，扎实开展投资环境集中整治行动，坚决打击强买强卖、强包强揽等影响投资环境的行为。建立项目跟踪服务机制，实现服务无距离、零障碍。加强重点领域安全监管，保障人民群众生命财产安全。
    提升生态文明。坚持资源节约型、环境友好型发展，推进国家森林城市创建。加大植树造林力度，创建更多的森林县区、森林城镇和森林村庄。开展全国水生态文明城市试点建设。加快沟河湖塘水系生态综合治理，启动姚家湾湿地水质改善项目，建成3个污水处理工程。开展大气环境综合整治，加强建筑施工、道路运输、企业料场、裸露煤场等扬尘治理，推进秸秆综合利用，全面完成脱硫脱硝、燃煤锅炉整治、“黄标车”淘汰任务，力争可吸入颗粒物下降10%以上。实施重点节能和资源综合利用项目50个以上。加强农村环境综合整治，推进矿区生态修复，为淮南创造更多的绿色财富，让群众享受更多的生态福利。
    三、加强法治政府建设
    各位代表，建设法治政府，必须牢固树立法治思维，忠实履行宪法和法律赋予的职责，学法尊法守法用法，着力营造“风清气正、干事创业”的良好政治生态。
    营造维权用法、遇事找法、办事依法的法治环境。全面贯彻依法治市实施意见，深入开展法治宣传教育，争创省法治城市创建活动先进单位，推进法治县区等创建。把全民普法和守法作为基础性工作，完成“六五”普法验收，推动全社会树立法治意识。优化基层法律服务布局，构建覆盖城乡的公共法律服务体系，加强困难群众法律援助。扎实开展“践行‘三严三实’、优化政治生态”学习讨论落实活动，切实维护好人民群众的合法权益。
    健全依法决策、科学决策、民主决策的决策程序。建立决策终身责任追究制度及责任倒查机制，把公众参与、专家论证、风险评估、合法性审查、集体讨论决定作为重大行政决策的法定程序，确保决策制度科学、程序正当、过程公开、责任明确。凡未经合法性审查或审查不合法的事项，不得提交讨论，不得发布施行。建立健全决策、执行、监督、评估、反馈闭环机制。全面推进政务公开，做到决策公开、执行公开、管理公开、服务公开和结果公开，提高决策的透明度。
    恪守依法行政、执法必严、违法必究的工作准则。自觉维护宪法和法律权威，坚持法定职责必须为、法无授权不可为。加强干部教育、培养、管理，强化公务员队伍素质，提升依法履职能力，争做法治型干部。扩大行政立法公众参与范围，制定政府规章2件。普遍建立政府法律顾问制度。严格执法人员持证上岗和资格管理制度，探索建立执法过程记录制度。合理配置执法力量，重点在安全生产、食品药品安全、资源环境、交通运输等领域推行综合执法。落实行政执法责任制，确保严格规范公正文明执法。
    完善权力运行、权力监督、权力制约的体制机制。自觉接受人大法律监督、政协民主监督和司法监督、社会监督、舆论监督，认真办理人大代表议案建议和政协委员提案。加大审计监督力度，加强公共资金、国有资源、国有资产等领域审计。强化行政监察，保障监察部门依法独立行使职权。严格落实党风廉政建设各项规定，确保资金安全、工程安全、生产安全和干部安全。持续聚焦“四风”问题，全面整治“吃空饷”行为，确保“三公”经费只减不增。严格落实“一岗双责”，坚决实行“一案双查”，不断提高拒腐防变和抵御风险的能力。坚持勤政廉政相统一，想干事、干成事、不出事，努力取得经得起实践、群众和历史检验的业绩。
    各位代表，新常态需要新作为，新作为开创新局面。让我们紧密团结在以习近平同志为核心的党中央周围，在中共淮南市委的坚强领导下，团结依靠全市人民，共同创造淮南更加美好的未来！
